# Development and validation of a patient-centered communication scale for nurses

**DOI:** 10.1186/s12912-024-02174-7

**Published:** 2024-08-13

**Authors:** Youngshin Joo, Yeonsoo Jang, Chang Gi Park, You Lee Yang

**Affiliations:** 1 College of Nursing and Brain Korea 21 FOUR Project, Yonsei University, Seoul, Republic of Korea; 2 College of Nursing and Mo-Im Kim Nursing Research Institute, Yonsei University, Seoul, Republic of Korea; 3 College of Nursing, University of Illinois Chicago, Chicago, IL USA; 4 College of Nursing, Eulji University, 553, Sanseong-daero, Sujeong-gu, Seongnam-si, Gyeonggi-do 13135 Republic of Korea

**Keywords:** Patient-centered care, Communication, Nurses, Factor analysis, Psychometrics

## Abstract

**Background:**

Patient-centered care aims to prevent disease and promote well-being by actively involving patients in treatment and decision-making that is based on respecting the patients and their families. However, no scales have been developed to assess patient-centered care from the nurse’s perspective. This study aimed to develop a scale to measure nurses’ level of patient-centered communication and confirm its validity and reliability.

**Methods:**

A methodological cross-sectional study was adopted to develop and validate the Patient-Centered Communication Scale (PCCS). The items were developed through a literature review and online interviews with nurses. Content validity was assessed by experts and the content validity index was calculated. A pretest of the questionnaire was conducted with 10 clinical nurses. To evaluate the factor structure and internal consistency reliability, the PCCS was administered online to 325 nurses in South Korea. Data were analyzed using descriptive statistics, explanatory factor analysis (EFA), and confirmatory factor analysis (CFA).

**Results:**

The final instrument consisted of 12 items and three factors: (1) information sharing, (2) patient-as-person, and (3) therapeutic alliance. EFA revealed a distinct three-factor structure, explaining 59.0% of the total variance. CFA confirmed the adequacy of the model fit and validated the inclusion of the final items. The Cronbach’s alpha values ranged from 0.60 to 0.77, indicating acceptable internal consistency. Convergent validity was evidenced by the correlation between the PCCS and a measure of interpersonal communication competence.

**Conclusions:**

The 12-item PCCS showed good reliability, construct validity, and convergent validity. The scale has utility for measuring the level of patient-centered communication skills in nurses.

**Supplementary Information:**

The online version contains supplementary material available at 10.1186/s12912-024-02174-7.

## Background

Communication is a vital component for nurses to establish therapeutic relationships with patients and their families, as well as to maintain cooperative relationships with other healthcare providers [1]. According to the Beryl Institute, key elements in the nursing experience of consumers include effective communication, which encompass active listening and clear conversations between healthcare providers and patients, and using understandable language when interacting with patients and their families [2]. However, in clinical practice, nurses often encounter challenges in communicating with patients, their families, and other healthcare providers. These difficulties not only diminish the quality of care but also elevate the risk of medication errors, potentially resulting in adverse outcomes or even fatalities [3]. Recent studies have shown that improved communication skills among nurses correlate with reduced medical errors, enhanced quality of care, better nursing performance, increased job satisfaction, stronger organizational commitment, and higher self-efficacy [2, 4, 5].

Current trends in the healthcare system emphasize the importance of nurses providing patient- and family-centered care (PFCC). This approach represents a philosophical shift in medical care, emphasizing collaboration between healthcare providers and patients along with their families [6]. PFCC represents the ideal direction for healthcare systems and policies, as it promotes active involvements of patients and their families in decision-making based on their values, preferences, and needs [7]. Previous studies have emphasized nurses’ communication competency as the primary factor in performing PFCC. Additionally, communication has been identified as a significant associating factor [8, 9]. To align with this trend, a number of communication programs have been developed by healthcare providers to enhance PFCC, such as the situation, background, assessment, recommendation (SBAR) tool and assertiveness training programs for nurses [10]. These programs not only improve communication skills, the level of clinical performance, critical thinking skills, job satisfaction, and self-efficacy but also reduce turnover intention in nurses [11, 12].

In clinical practice, nurses’ communication should be goal-oriented and focus on enhancing patients’ physical and mental health, which is consistent with patient-centered communication (PCC) [13]. PCC aims to prevent disease and promote well-being by actively involving patients in their treatment and decision-making that is based on respecting patients and their families [14]. However, there is no study evaluated nurses’ level of PCC, who communicate the most with patients and their families in clinical settings. Furthermore, the commonly used scales to measure PCC were not developed for clinical nurses.

Thus far, the scales developed for measuring general communication skills or for communication competencies of nursing students in simulation courses have been used [11, 15]. For example, Hur [15]’s global interpersonal communication competence scale is often utilized to assess clinical nurses’ communication skills, although it was not designed specifically for nurses in clinical settings. Some PCC scales that assess the patient’s perspective have been developed [16, 17]. Moser et al. [16]'s PCC scale evaluated patients' experiences of PCC, while Reeve et al. [17] validated communication measures specifically from colorectal cancer patients. However, these instruments solely reflect the patient’s viewpoint and not that of the providers. There exists a instrument primarily developed by researchers for their own studies that evaluates nurses’ perspectives, yet it has not been validated [18]. Furthermore, another instrument evaluates nurses' attitudes towards patients communication [19], while a tool specifically assesses PCC in older patients with acute myeloid leukemia [20], limiting its applicability. Moreover, these existing instruments have limitations in evaluating PCC from the perspective of nurses, as they do not fully capture the attributes of the therapeutic relationship between nurses and patients. Therefore, it is necessary to develop and analyze the psychometric properties of an instrument that comprehensively measures PCC in nurses.

### Purpose

This study aimed to develop the Patient-Centered Communication Scale (PCCS) for clinical nurses and evaluate the reliability and validity of the scale.

## Methods

### Study design

This was a methodological cross-sectional study to develop and validate the PCCS for nurses.

### Scale development

#### Conceptual framework of the Patient-Centered Communication Scale (PCCS)

In this study, the concept of PCC was defined as a set of actions aimed at empowering patients or their families, including providing adequate information, addressing emotions, expressing empathy, actively reflecting their values and preferences in decision-making, and involving them in therapeutic decisions. We adopted five domains from the PCC model [14]: biopsychosocial, patient-as-person, sharing power and responsibility, therapeutic alliance, and provider-as-person.

The biopsychosocial domain encompasses the biomedical, psychological, and sociological aspects of illness, emphasizing information exchange for patients and families. The patient-as-a person domain involves understanding patients’ unique personalities beyond their illness. The sharing power and responsibility domain includes actively engaging the patient or family members in treatment decision-making and reaching an agreement on the care plan. The therapeutic alliance domain focused on the quality of the relationship between healthcare providers and patients. Mutual understanding of treatment goals, a personal bond, or a patient's trust or perceptions of healthcare providers can influence the quality of the relationship. The provider as-a-person domain involves effectively engaging with other healthcare providers in patients’ care or treatment and recognizing one’s own emotions experienced while caring patients [14, 21].

#### Preliminary items of the PCCS

To develop the preliminary items of the PCCS, the literature reviews and e-mail interview were conducted. Firstly, we searched PubMed, the Cumulative Index to Nursing & Allied Health Literature, the Research Information Sharing Service, and the Korean-Studies Information Service System databases using the keywords such as “patient-centered communication,” “therapeutic communication,” “communication skill,” and “nurs*. Through this literature review, we developed initial 50 items.

We conducted an e-mail interview consisting of four open-ended questions regarding the concept of PCC, distinctions between PCC and non-PCC approaches, specific methods or strategies for implementing PCC, and the essential competencies for nurses to effectively engage in PCC. For interviews, we selected 10 nurses using purposive sampling, as suggested by Sandelowski [22], which is considered adequate for qualitative study when contextual factors among participants are similar. Inclusion criteria were bedside nurses in hospitals with over 300 beds, with a minimum of one year of clinical experience caring for patients. Exclusion criteria were nurses with less than one year of hospital experience, nurse managers or those not directly involved in bedside patient care.

We distributed the questions to 10 nurses using their individual e-mail addresses and received responses. The collected data were analyzed using content analysis [23]. To analyze the content, we collected descriptions from 10 nurses for each item and thoroughly reviewed them, selecting meaningful statements and grouping similar responses together. After reviewing the grouped content and holding team meetings, we established categories based on five domains of PCC and added 20 items.

According to the results of the literature review and e-mail interviews, a total of 70 items were created. These items were then classified by the first author according to the five domains of the PCC concept and later discussed within the research team. Using a team to develop items benefits from the fact that people express similar ideas in diverse ways [24].

Subsequently, during research team meetings, we refined the classification and removed 19 items. This included: 1) items with duplicate or similar meanings, especially those related to providing information to patients and their families, engaging them in care processes, and demonstrating respect for their needs, values, and preferences; and 2) items that were not directly relevant to PCC by nurses. Consequently, 51 items were selected as the preliminary items for the PCCS.

#### Content validity

Experts were used to evaluate the content validity of the PCCS. Four clinical nurses with more than 10 years of work experience and three nursing professors participated as the expert panel. Following the recommendation of Burns and Grove (2005), seven experts were recruited to assess the content validity of the PCCS [25]. The expert panel consisted of four nurses and nurse managers with over 10 years of clinical experience and have master’s degrees or higher. Additionally, three nursing professors who have experience in communication-related education and research, as well as scale development research, were included.

A 4-point Likert scale was used for the evaluation of the content and to determine the item content validity index (CVI) (I-CVI; higher than 0.80) and scale CVI (S-CVI; higher than 0.90) [26]. The response options were categorized based on the degree of agreement with the content validity of each item, ranging from 'Not valid at all' (1-point) to 'Very valid' (4-points). Additionally, experts were asked to provide reasons for any item rated 2 points or lower, as well as overall opinions on items that needed to be revised, deleted, or added. Furthermore, based on the experts’ feedback, we had research team meetings to discuss, modify, integrate, and extract items. Items that were ambiguous or confusing were removed from the preliminary scale.

The I-CVI ranged from 0.57 to 1.0, and 13 items had a value less than 0.80. The S-CVI average was 0.86. Based on the experts’ feedback and study team meetings, 11 of the 13 items with a CVI of less than 0.80 were deleted [26]. The deleted items were difficult to link directly to communication and were highly abstract. However, the two items that had a value less than 0.80 were retained being modified. Furthermore, based on the feedback from the experts, items that were 0.80 or higher and had duplicate content were integrated into one item. Through this process, we developed a 31-item questionnaire.

#### Pretest and cognitive testing

Based on a previous study that developed an instrument for nurses [27], a pretest of the 31-item questionnaire was conducted with 10 bedside clinical nurses to evaluate the understandability of the items, the time it took to complete the questionnaire, the appropriateness of the arrangement, and the length of each item. Then, among the 10 nurses who participated in the pretest, five were selected for telephone interviews to identify the items. Then, among the 10 nurses who participated in the pre-test, five were selected for telephone interviews to identify the items. Based on feedback suggesting that providing more specific examples or clarifying ambiguous terms would enhance clarity, we revised the items accordingly. It took 9.40 ± 3.86 min to complete the questionnaire. The understandability of the items, appropriateness of the items’ arrangement, and time to complete the questionnaire received more than 3 out of 4 points. The expressions of three items were modified in keeping with the participants’ feedback.

### Validation of the PCCS

#### Study participants

The study participants were bedside nurses working at hospitals in South Korea. The inclusion criteria were nurses (1) working at a hospital with more than 300 beds, (2) who had at least one year of work experience, and (3) working as a bedside nurse. Exclusion criteria were nurses (1) who were working at a specialized hospital, such as dentistry, oriental medicine, or nursing home, or (2) who were a charge nurse or unit manager.

To evaluate construct validity using exploratory factor analysis (EFA), a sample of five times the minimum number of items is required [28] and a confirmatory factor analysis (CFA) requires a minimum sample size of 150 participants [29]. The preliminary PCCS consisted of 31 items; therefore, 155 participants were needed for the EFA and 150 for the CFA. Considering a 90% valid response rate, a total of 339 nurses were recruited. After excluding questionnaires that had duplicate or inappropriate answers, questionnaires from 325 participants were included in the analysis.

### Instruments

#### PCCS

The PCCS consisted of 31 items that participants responded to using a 5-point Likert scale, where 1 = “strongly disagree” and 5 = “strongly agree.” The scale was constructed with eight items for the biopsychosocial domain, nine items for patient-as-person, three items for sharing power and responsibility, four items for therapeutic alliance, and seven items for the provider-as-person. Higher scores indicate a higher level of PCC.

#### Interpersonal communication skills

To evaluate convergent validity, we used the Global Interpersonal Communication Competence (GICC) Scale [15], which is based on Rubin’s [30] eight communication skills (self-disclosure, empathy, social relaxation, assertiveness, interaction management, expressiveness, immediacy, supportiveness), and seven more concepts (concentration, goal detection, noise control, efficiency, assertiveness, conversational coherence, responsiveness) on interpersonal communication. Participants responded to the 15 items using a 5-point Likert scale. Higher scores indicate a higher degree of interpersonal communication skills. Hur [15] reported a Cronbach’s alpha of 0.74 for the scale.

#### General and professional characteristics

Data were collected through self-report on the following participant characteristics: gender, age (year), education, marital status, religion, work experience (year), and work unit.

#### Data collection

Data were collected through online survey via the Survey Monkey website from June 27, 2022 to July 15, 2022. A recruitment notice that included a URL address to participate in the study was posted in two online communities freely opened to the nurses across the country. After accessing the URL link, potential participants were asked to read the informed consent form, and those who decided to participate in the study clicked on “agree” to go to the next page. Then, participants were asked about each inclusion/exclusion criterion, ensuring that only those who answered "yes" to all criteria were eligible to participate in the survey. We permitted only one IP address to submit a single response to minimize duplicate participation. All the questions required a response to prevent missing values. We distributed an online coffee coupon to the participants who completed the questionnaire.

#### Data analysis

The data were analyzed using IBM SPSS/Win 28.0 (IBM Corp., Armonk, NY, USA) and R version 4.2.1 (R Foundation for Statistical Computing, Vienna, Austria). The process included the following steps.

1. The characteristics of the participants were analyzed using descriptive statistics, including frequency, percentage, mean, and standard deviation. Differences between the CFA and EFA groups were analyzed using the chi-square test and independent t-test.
2. An EFA was performed using principal axis factoring to extract meaningful structures common to all the items. We performed direct oblimin rotation to minimize the inter-factor correlations and make the factor more interpretable [31]. The Kaiser-Meyer-Olkin (KMO) test and Bartlett’s test for sphericity were conducted to determine whether the collected data were suitable for factor analysis.
3. A CFA was performed to test the accuracy of the structure revealed by the EFA; weighted least squares means and adjusted variance (WLSMV) were used. WLSMV is known as an appropriate estimation method to conduct CFA for Likert scales, with fewer than five points in medium-to-small sample sizes [32, 33]. Absolute fix and incremental fit indices were evaluated. The fit indices included χ^2^ statistic (*p*-value), normed χ^2^ (chi-square/degrees of freedom [CMIN/df]), goodness of fit index (GFI), standardized root mean square residual (SRMR), root mean square error of approximation (RMSEA), Tucker-Lewis index (TLI), and comparative fit index (CFI). The reference values for each fitness index were *p* > 0.05, χ^2^(CMIN/df) ≤ 3.0, GFI ≥ 0.90, SRMR ≤ 0.08, RMSEA ≤ 0.08, TLI ≥ 0.90, and CFI ≥ 0.90.
4. The Pearson correlation coefficient for the PCCS and GICC scale [16] was calculated to verify convergent validity.
5. Cronbach’s alpha coefficient was analyzed to evaluate the internal consistency reliability of the scale.

#### Ethical consideration

We received the approval of the Institutional Review Board of Eulji University prior to the study (IRB No. EUIRB2022-013).

All study participants were informed about the aims and the method of the study, and they were asked to provide written informed consent.

## Results

### Characteristics of the participants

A total of 325 bedside nurses participated in the study (175 for the EFA and 150 for the CFA). Most of the participants were women (95.4%), and the mean age was 32.15 ± 3.93 years. Most participants had a bachelor’s degree (86.2%), followed by a diploma (9.2%), or master’s degree or higher (4.6%). Mean years of work experience was 6.25 ± 3.55 years, and the working units were medical (37.8%), surgical (29.5%), obstetrics gynecology, pediatrics (14.5%), intensive care (11.1%), and emergency room (6.2%). There were no significant differences in gender, age, education, marital status, religion, or the working units between the EFA and CFA groups (Table 1).

**Table 1:** Demographic characteristics of participants (*n* = 325)

Variables	Categories	Total (*n* = 325)	EFA group (*n* = 175)	CFA group (*n* = 150)	x^2^	*p*
		**n(%) or M ± SD**				
Gender	Man	15 (4.6%)	10 (5.7%)	5 (3.3%)	1.040	0.308
	Woman	310 (95.4%)	165 (94.3%)	145 (96.7%)		
Age (year)		32.15 ± 3.93	32.15 ± 3.94	32.15 ± 3.92	0.111	0.739
	< 30	83 (25.5%)	46 (26.3%)	37 (24.7%)		
	≥ 30	242 (74.5%)	129 (73.7%)	113 (75.3%)		
Education	College	30 (9.2%)	18 (10.3%)	12 (8%)	1.653	0.437
	University	280 (86.2%)	147 (84%)	133 (88.6%)		
	≥ Master’s degree	15 (4.6%)	10 (5.7%)	5 (3.4%)		
Marital status	Married	153 (47.1%)	84 (48%)	69 (46%)	0.137	0.934
	Unmarried	170 (52.3%)	90 (51.4%)	80 (53.3%)		
	Divorced/Separated	2 (0.6%)	1 (0.6%)	1 (0.7%)		
Religion	Christian	74 (22.8%)	43(24.6%)	31 (20.7%)	2.108	0.550
	Catholic	32 (9.8%)	14(8%)	18 (12%)		
	Buddhist	31 (9.5%)	18(10.3%)	13 (8.6%)		
	No religion	188 (57.9%)	100(57.1%)	88(58.7%)		
Work experience (year)		6.25 ± 3.55	6.30 ± 3.60	6.20 ± 3.49	2.905	0.580
	1—3	56 (17.2%)	32 (18.3%)	24 (16%)		
	3 <—6	112(34.5%)	58 (33.1%)	54 (36%)		
	≥ 6	157(48.3%)	85 (48.6%)	72 (48%)		
Work unit	Surgical	96 (29.5%)	56 (32%)	40 (26.7%)	2.432	0.876
	Medical	123 (37.8%)	62 (35.4%)	61 (40.7%)		
	OB&GY, Pediatrics	47 (14.5%)	24 (13.7%)	23 (15.3%)		
	Intensive care	36 (11.1%)	20 (11.4%))	16 (10.6%)		
	Emergency	20 (6.2%)	11 (6.3%)	9 (6.0%)		
	Other	3 (0.9%)	2 (1.2%)	1 (0.7%)		

*EFA* Exploratory factor analysis, *CFA* Confirmatory factor analysis, *OB* Obstetric, *GY* Gynecology, *M* Mean, *SD* Standard deviation

### Item analysis

The item analysis revealed the mean score for the 31 items was 3.73 ± 0.48, and the range was 3.42–4.03 (standard deviation 0.71–1.01). Skewness and kurtosis were less than ± 2.00 on all the items, so normality was satisfied. The item-total correlations were more than 0.30 and less than 0.80, implying that no items deviated from the standard (see Supplementary file 1) [34].

### Exploratory factor analysis

The KMO value of the data was 0.87, and Bartlett’s test of sphericity was χ^2^ = 632.95 (*p* < 0.001); therefore, the conditions for the EFA were satisfied. After checking repeatedly for communality and factor loadings, three factors with an eigenvalue of 1.0 or greater were derived, and 16 items were deleted. The reasons are those items exhibited less than 0.40 in communality or cross loaded on two or more factors and the difference in the factor loading value was less than 0.20. The standard criteria were set at 0.40 [35], and prior study on scale development for nurses [36]. Additionally, three items did not load on any factor. Consequently, 12 items were confirmed and accounted for 59.0% of the variance. Factor 1 (information sharing) comprised five items and explained 38.2% of the variance. Factor 2 (patient-as-person) comprised four items and explained 10.6% of the variance. Factor 3 (therapeutic alliance) comprised three items and explained 9.8% of the variance (Table 2).

**Table 2:** Rotated factor pattern matrix of EFA (*n* = 175)

No	Items	Factor		
		**1**	**2**	**3**
**4**	Identify the information patient/families want to know and provide it in a timely manner	**.816**	.188	.215
**9**	Check the patient or family’s level of understanding after providing the information	**.790**	.344	.424
**5**	Provide enough time to ask questions	**.697**	.391	.254
**1**	Describe the medical treatment/procedure or nursing intervention	**.619**	.409	.686
**2**	Describe the reason for the necessity of medical treatment/procedures or nursing intervention	**.516**	.337	.667
**7**	Use open-ended questions to allow the patient and family to express their feelings, needs, and opinions freely (e.g., “How are you feeling today?”)	.107	**.826**	.278
**13**	Encourage the patient or family to express any feelings they may experience related to the illness	.281	**.732**	.428
**12**	Look at the situation from the patient or family’s point of view and communicate with them	.418	**.711**	.148
**8**	Listen actively to the words of the patient and family members when communicating	.404	**.658**	.361
**28**	Communicate and share my negative and positive emotions while providing care with my fellows and/or medical staff (e.g., physician, fellow nurse, etc.)	.277	.311	**.776**
**23**	Introduce departments or other organizations that can help the patient or family (e.g., social work team, caregiver association, psychotherapy etc.)	.249	.459	**.718**
**22**	Collaborate across disciplines (e.g., physicians, nutritionist, physical therapist, etc.) to establish therapeutic interventions and aid patient and/or family’s understanding	.162	.155	**.694**
Eigen value		4.580	1.277	1.175
Explained variance (%)		38.169	10.643	9.788
Total explained variances (%)		38.169	48.811	58.599

*EFA* Exploratory factor analysis

### Confirmatory factor analysis (CFA)

A CFA was performed on the three factors and 12 items of the PCCS. All items had a standardized factor loading of 0.40 or higher. The fitness indices were as follows: χ^2^ = 57.601 (*p* < 0.001), CMIN/df = 1.12, GFI = 0.98, SRMR = 0.05, RMSEA = 0.03, TLI = 0.98, CFI = 0.98. All the fit indices satisfied the criteria (Table 3).

**Table 3:** Findings of the confirmatory factor analysis (*n*=150)

Factor		Item	Standardized estimates		SE	z-value		*p*
**1**		4	0.491					
		9	0.554		0.27	4.677		< .001
		5	0.558		0.30	4.696		< .001
		1	0.734		0.36	5.362		< .001
		2	0.720		0.22	5.325		< .001
**2**		7	0.631					
		13	0.604		0.16	5.784		< .001
		8	0.570		0.17	5.537		< .001
		12	0.691		0.16	6.344		< .001
**3**		28	0.480					
		23	0.421		0.24	3.899		< .001
		22	0.551		0.27	4.615		< .001
**Fitness index**	**X**^**2**^**(*p*)**	**df**	**CMIN/df**	**GFI**	**SRMR**	**RMSEA (90% CI)**	**TLI**	**CFI**
**Criteria**	(> .05)		≤ 3	≥ 0.90	≤ 0.08	≤ 0.10	≥ 0.90	≥ 0.90
**Model**	57.601 (*p* = 0.24)	51	1.12	0.984	0.045	0.029	0.980	0.984

*CFI* Comparative fit index, *CMIN* Chi-square, *df* degree of freedom, *GFI* Goodness of fit index, *SRMR* Standardized root mean-square residual, *RMSEA* Root mean square error of approximation, *SE* Standard error, *TLI* Turker-lewis index

### Convergent validity

The correlation coefficient between the PCCS and GICC scale was 0.68 (*p* < 0.001), providing support for convergent validity.

### Reliability

The Cronbach’s alpha of the total scale was 0.84. The Cronbach’s alpha for the three factors were 0.77, 0.72, and 0.60 (Table 4).

**Table 4:** Reliability coefficients

Factor	Item	Mean ± SD	Cronbach’s alpha if item deleted	Cronbach’s alpha
**1 (information sharing)**				**0.77**
	1	3.42 ± 1.01	0.70	
	2	3.77 ± 0.78	0.72	
	4	3.94 ± 0.83	0.75	
	9	3.76 ± 0.90	0.72	
	5	3.65 ± 0.95	0.74	
**2 (patient-as-person)**				**0.72**
	7	3.63 ± 0.88	0.65	
	13	3.84 ± 0.84	0.67	
	8	3.78 ± 0.84	0.65	
	12	3.77 ± 0.88	0.68	
**3 (therapeutic alliance)**				**0.60**
	28	3.66 ± 0.85	0.44	
	23	3.55 ± 0.92	0.53	
	22	3.68 ± 0.90	0.48	
**Total**	**12-item**	3.73 ± 0.78		**0.84**

*SD* Standard deviation

## Discussion

This study aimed to develop and validate a Patient-Centered Communication Scale (PCCS) for clinical nurses, comprising three factors and 12 items. The PCCS demonstrated good reliability and validity as a measure of PCC in clinical nurses. The scale could be considered as a useful tool for evaluating and designing improvements of clinical nurses’ skill of PCC. This study developed the scale based on the domains of PCC, which including biopsychosocial, patient-as-person, sharing power and responsibility, therapeutic alliance, and provider-as-person [14].

Regarding the validity of the instrument, study findings showed an adequate content validity for the questionnaire. Thus, the items that make up the instrument accurately and clearly reflect what it is meant to measure and the domain in which it is meant to measure. In terms of construct validity, factor analysis was conducted. In the EFA, 12 items and three dimensions were extracted. The factor loadings of the items were all greater than 0.4. In the CFA, a well-fitting model was obtained, with all indices in the acceptable range. The three-factor structure of the scale had an appropriate fit.

The first subscale extracted in the EFA was the information sharing, which is consistent with the biopsychosocial perspective domain in the PCC conceptual framework [14]. The factor consists of five items and has the most explanatory power among the three factors at 38.17%. The items are related to explaining interventions and procedures about medical treatment and nursing care. The Institute of Patient- and Family-Centred Care (IPFCC) emphasized the importance of delivering information which is timely, complete, and accurate [37]. According to these items, the biopsychosocial domain was a large portion of nurses’ communication with patients and their families, primarily addressing acute biomedical issues such as vital signs, technical matters, medical history, and nursing interventions [14]. Therefore, it is an essential component and considered as one of the core concepts in PCC. Additionally, this factor consists of items wherein nurses assess patients’ and families’ understanding and allowing them sufficient time to ask questions. This emphasizes the bidirectional communication of patient-centered care. Furthermore, patients are not solely providing information about their medical symptoms and illness, they are actively participating in a reciprocal exchange of disease-related information [16].

The second subscale of the PCCS was patient-as-person, which has four items. This factor is consistent with the patient-as-person domain in the PCC conceptual framework, which emphasizes the aspects of caring that include sharing emotions and understanding individual worries and concerns related to the disease rather than focusing only on curing the disease [14]. Open ended questions are used to encourage patients and families to express their emotions. PCC includes eliciting the patient’s agenda through open-ended questions and engaging in focused active listening. The key features of PCC involve understanding the patient’s perspective on the illness and demonstrating empathy [38]. This understanding encompasses exploring the patient’s feelings, ideas, concerns, and experiences related to the impact of the illness, as well as recognizing the patient’s expectations from health care providers [39].

Non-verbal communication is critically important to express empathy and achieve patient-centered communication [40]. However, some preliminary items in this study about using non-verbal communication methods (e.g., touch and non-verbal expressions of empathy) and casual conversation with patients were deleted during factor analysis due to their low communality. This finding may be derived due to barriers on patient-centered care and communication. In previous studies, nurses spent less time on interactions with patients due to poor staffing ratios, higher workload, and lack of institutional and healthcare system support [1, 8]. This may be reflected in the relatively lower performance of PCC, such as having a daily conversation with a patient or understanding their feelings, because nurses frequently experience a great amount of pressure to accomplish a large amount of work within their duty hours [41, 42].

The third subscale of the PCCS was therapeutic alliance, which is consistent with the therapeutic alliance and provider-as-person domains in the PCC conceptual framework [14]. The factor consists of three items regarding introductions of other departments or organizations to the patient or family, collaborating across disciplines, and communicating and sharing emotions with colleague. However, in the PCCS, 3 items were correlated to alliance between healthcare providers, without including collaboration with patients and their families. According to the IPFCC, alliance refers not only to the care of patients and families, but also to health care providers involved in the development, implementation, and evaluation of policies beyond patients’ basic care [37]. Despite this expanded definition, there remains a lack of consensus between patients and healthcare providers [43], and Interprofessional collaboration among healthcare providers is more commonly addressed than collaboration between patients and healthcare providers in clinical settings [44]. This alliance ultimately occurs as a result of patient centered communication and enhances the quality of patient-and family-centered care [43]. Further studies are recommended for clearly establishing the concept and strategies to enhance the alliance between patients and health care providers.

Two domains, provider-as-person and sharing power and responsibility, were conceptually validated but did not ultimately emerge as final domains; corresponding items were removed through the EFA process. Following clinical nursing circumstances likely influenced the exclusion of these domains from the final PCCS.

The provider-as-person domain, which assesses healthcare providers' self-evaluation of communication skills and participation in training programs, was removed. Nurses faced challenges in dealing with aggressive patients, communicating with seriously ill patients, and encountered barriers to training participation [45]. To address this, tailored communication education programs addressing actual clinical difficulties and incentive strategies for participation should be adopted for nurses.

Another excluded domain, sharing power and responsibility, promotes mutual equality between healthcare providers and patients, emphasizing active patient involvement in decision-making and consideration of their personal experiences [14, 21]. However, nurses often felt distant from such interactions, deferring decisions to doctors perceived as having superior knowledge [46]. Additionally, nurses believed this domain was more prominent between patients, families, and physicians rather than nurses [15].

In this study, 70 initial items were developed; however, only 12 items were finally confirmed as part of PCCS. Initial items were developed to encompass a broad range of attributes related to PCC, with the aim of distinguishing them from existing PCC instruments and to maximize coverage of therapeutic relationships between patients and nurses in clinical settings. Despite recognizing PCC as crucial component in healthcare, paternalistic values persist, and nurses may lack perception of collaboration among healthcare providers, patients, and families. Various clinical situations and factors, including nurses' personal values and workplace culture, could influence the study results [44]. We recommend developing additional items for removed domains and conducting validity testing again.

Convergent validity was assessed by analyzing the correlation with GICC and PCCS, which yielded a value of 0.68. Based on the criterion that a correlation coefficient between 0.40 and 0.80 ensures convergent validity [47], this value was considered to be sufficient. The reliability was assessed using Cronbach's alpha for all the items, resulting in 0.84, and each subscale ranged from 0.60 to 0.77. Cronbach's alpha tends to decrease with fewer items, and the therapeutic alliance scale, comprised of three items, exhibited the lowest alpha value of 0.60. However, for both the overall scale and each subscale, values were above the criterion of 0.60 [47], indicating satisfactory internal consistency and reliability of the instrument.

This study has several limitations. Firstly, this study used convenience sampling, which may have contributed to sampling bias. Secondly, during the process of obtaining content validity from experts, we did not send revised items back for a second round of feedback. Re-evaluation could enhance the robustness of initial item development. Therefore, it may be necessary to consider incorporating this step in future tool development. Thirdly, the reliability coefficient of the therapeutic alliance factor is low, possibly due to the small number of items. Therefore, the reliability requires further evaluation using larger sample size. Fourthly, as this study was conducted in South Korea, cultural background may have influenced the study results. Therefore, further study is recommended to test the psychometric properties of the PCCS in various countries.

## Conclusion

The PCCS developed in this study showed good validity and reliability. The scale consists of three factors and 12 items: information sharing (five items), patient-as-person (four items), and therapeutic alliance (three items). The scale can be used to assess the level of PCC in nurses, increase nurses’ understanding of PCC in a hospital setting, and promote patient-centered care. Further studies are recommended to test the validity of the PCCS in various hospital settings with a larger sample size and to explore factors associated with PCC in nurses.

### Supplementary Information

Supplementary Material 1.

## Data Availability

All data generated or analyzed during this study are included in this article. The data can be made available upon reasonable request from the corresponding author.
