# Supplementary material for: Development and validation of a patient-centered communication scale for nurses
Source: BMC Nurs. 2024 Aug 13;23:550. doi: 10.1186/s12912-024-02174-7 (PMC11320938; doi:10.1186/s12912-024-02174-7)
Supplement: Supplementary file 1 — Supplementary Material 1. [file 12912_2024_2174_MOESM1_ESM.docx]

**Supplementary file 1**

The result of item analysis

| Items | M | SD | Skewness | Kurtosis | Item-total  correlation |
| --- | --- | --- | --- | --- | --- |
| Item 1. | 3.42 | 1.01 | -0.22 | -1.10 | 0.69 |
| Item 2. | 3.77 | 0.78 | -0.24 | -0.30 | 0.61 |
| Item 4. | 3.94 | 0.83 | -0.42 | -0.40 | 0.52 |
| Item 5. | 3.65 | 0.95 | -0.50 | -0.32 | 0.59 |
| Item 7. | 3.63 | 0.88 | -0.48 | -0.24 | 0.52 |
| Item 8. | 3.84 | 0.84 | -0.45 | -0.10 | 0.55 |
| Item 9. | 3.76 | 0.90 | -0.55 | -0.13 | 0.61 |
| Item 10. | 3.89 | 0.89 | -0.56 | -0.19 | 0.60 |
| Item 13. | 3.70 | 0.95 | -0.51 | 0.00 | 0.57 |
| Item 22. | 3.68 | 0.90 | -0.41 | -0.34 | 0.48 |
| Item 23. | 3.55 | 0.92 | -0.20 | -0.69 | 0.54 |
| Item 28. | 3.66 | 0.85 | -0.28 | -0.35 | 0.54 |

Note. M:Mean; SD:Standard deviation
